# In vivo cytotoxicity analysis of bioceramic root canal sealers on zebrafish embryo

**DOI:** 10.34172/joddd.2023.39163

**Published:** 2023-12-30

**Authors:** Siti Aisyah Nadirah Ja'apar, Solachuddin Jauhari Arief Ichwan, Musliana Mustaffa

**Affiliations:** ^1^Department of Biotechnology, Kulliyyah of Sciences, International Islamic University Malaysia (IIUM), Jalan Sultan Ahmad Shah, Bandar Indera Mahkota, 25200, Kuantan, Pahang, Malaysia; ^2^Department of Fundamental Dental and Medical Sciences, Kulliyyah of Dentistry (KOD), IIUM, Jalan Sultan Ahmad Shah, Bandar Indera Mahkota, 25200, Kuantan, Pahang, Malaysia; ^3^Dentistry Programme, PAPRSB Institute of Health Sciences, Universiti Brunei Darussalam, Jalan Tungku Link, Gadong, BE1410, Brunei; ^4^Department of Restorative Dentistry, KOD, IIUM, Jalan Sultan Ahmad Shah, Bandar Indera Mahkota, 25200, Kuantan, Pahang, Malaysia

**Keywords:** Bioceramic root canal sealers, Cytotoxicity, Endodontics, In vivo, Zebrafish embryos

## Abstract

**Background.:**

This study evaluated the cytotoxicity of four bioceramic root canal sealers (RCSs) in vivo. The embryonic zebrafish characteristics, such as mortality, survival, hatching, and general morphology, served as the parameters for assessing cytotoxicity.

**Methods.:**

The RCSs, namely GuttaFlow Bioseal, MTA Fillapex, CeraSeal Bioceramic, and iRoot SP, were mixed according to the manufacturer’s guidelines. The extract solution was prepared by immersing the set RCS into 1X dilution of E3 solution. Then, the extract solution was delivered into a Petri dish where zebrafish embryos were allowed to develop. Cytotoxicity was evaluated 24, 48, 72, and 96 hours after fertilization.

**Results.:**

The Kruskal-Wallis test showed that except for GuttaFlow Bioseal, the mortality, survival, and hatching of zebrafish embryos for the remaining three bioceramic RCSs were significantly different from the negative controls (*P*<0.05). Significant differences were also evident in the mortality, survival, and hatching of zebrafish embryos between GuttaFlow Bioseal and three other RCSs (*P*<0.05).

**Conclusion.:**

GuttaFlow Bioseal was less cytotoxic than other bioceramics RCSs; MTA Fillapex, CeraSeal Bioceramic root canal sealer, and iRoot SP root canal sealer exhibited comparable cytotoxicity.

## Introduction

 Bioceramic root canal sealers (RCSs) have been used in endodontics for years. Their popularity has grown with the increased use of bioceramic technology in medicine and dentistry.^1^ The calcium silicate component of the bioceramic RCSs performs better than other conventional RCSs.^2^ Calcium silicate is a bioactive material that promotes hard tissue formation in the dental pulp and bone, potentially creating a hydroxyapatite layer with chemical constituents and structures similar to the bone.^3^ It potentially mimics human tissues while stimulating osteoinduction regeneration response.^4,5^ In endodontics, calcium silicate-based bioceramics interact with cells, affecting cellular proliferation, differentiation, and migration.^6^

 The first generation of bioceramics, mineral trioxide aggregate (MTA), takes longer to set with difficult handling characteristics^5^ and may discolor tooth structure because of its iron content.^6^ Hence, a new generation of bioceramics was developed. At present, commercially available RCSs include GuttaFlow Bioseal (Colténe/Whaledent AG, Altstatten, Switzerland), MTA Fillapex (Angelus, Londrina, Brazil), CeraSeal Bioceramic (Meta-Biomed, Korea), iRoot SP (Innovative BioCeramix Inc., Vancouver, Canada), EndoSeal MTA (Maruchi, Wonju, Korea), Tech Biosealer Endo (Isasan, Como, Italy), Sankin Apatite root canal sealer (Sankin Kogyo, Tokyo, Japan) and others.

 GuttaFlow Bioseal is a silicone-based product made of gutta-percha powder and polydimethylsiloxane with silver nanoparticles as a preservative. Silica, calcium oxide, and phosphorous oxide particles are also added to this sealer to enhance tissue regeneration activity.^7^ The inflammatory cells of GuttaFlow Bioseal have been evaluated using animal models, but the in vivo cytotoxicity remains unexplored. When implanted into the subcutaneous tissue of Wistar rats, GuttaFlow Bioseal showed mild inflammatory cells on day eight and no inflammatory cells after thirty days of the observation period.^4^ When various RCSs were evaluated, the inflammatory cells on day 30 showed mild inflammation with GuttaFlow Bioseal, GuttaFlow 2, and AH Plus.^4^

 MTA Fillapex consists of dicalcium and tricalcium silicate, MTA particles, resin (salicylate resin and diluted resin), and bismuth oxide as a radiopacifier.^8^ MTA Fillapex has good dimensional stability,^9^ solubility,^10^ flowability,^11^ and radiopacity.^12^ However, it did not meet the requirements of ISO with a longer setting time.^11^ When the MTA Fillapex was assessed using animal models, moderate inflammatory response on day 7 and almost no inflammatory response on days 15, 30, 60, and 90 were observed,^13^ indicating reduced inflammation over time. In contrast, MTA Fillapex showed a severe inflammatory response on day 90.^14^ Meanwhile, severe inflammatory responses were observed for MTA Fillapex on days 7 and 14, with a mild inflammatory response on day 30 and a trivial inflammatory response on day 60,^15^ indicating a reduction in inflammation over time. However, another study found that MTA Fillapex caused moderate inflammatory responses on days 7 and 30.^8^ Due to the differences in findings, in vivo cytotoxicity analysis is necessary to further explore this endodontic sealer.

 CeraSeal Bioceramic comprises calcium silicate, zirconium oxide, calcium hydroxide, and thickening agents.^16^ Few studies on the physicochemical properties of CeraSeal Bioceramic have been performed, including its solubility and flowability. According to the results, it does not meet the ISO specifications for RCS.^17^ Since no in vivo studies have evaluated CeraSeal Bioceramic, our understanding of the in vivo cytotoxic effects of this RCS is limited.

 iRoot SP is composed of calcium silicate, calcium phosphate, niobium oxide, zirconium oxide, and calcium hydroxide.^18^ Its dimensional stability, solubility, flowability, and radiopacity meet the ISO 6876 specifications.^9,11^ However, its setting time does not.^11^ When introduced into the dorsal subcutaneous connective tissue and tibias of Wistar rats, iRoot SP induced moderate infiltration of chronic inflammatory cells on day 7. In addition, a comparison of the infiltration of the inflammatory cells of iRoot SP root canal sealer to ProRoot MTA and AH Plus on day 60 showed mild infiltration of the inflammatory cells and no reactivity.^19^ Previous findings showed moderate inflammatory response for iRoot SP on days 7 and 15, with absent to moderate inflammatory response on day 30 and absent to mild inflammatory cells on day 90 of the observation period,^14^ indicating a reduction in inflammation over time. In addition, there was a severe inflammatory response for iRoot SP on day 7, a mild to moderate inflammatory response on day 30, and a mild inflammatory response on days 50 and 100.^20^ According to the findings of previous in vivo studies, iRoot SP is a potential bioceramic RCS for widespread use, with excellent biological response characteristics.

 Additionally, the in vivo cytotoxicity of this material has not been fully investigated. In contrast to the in vitro technique, the in vivo approach is less implemented, possibly because of more complex and time-consuming^21^ experimental conditions. However, this approach is worth exploring to support clinical investigations in the future. Mammalian cell culture assays have shown their efficacy in assessing the in vitro toxicity at various biological endpoints, including cell damage, cell growth, membrane effects, and the rate of cell proliferation.^22^ However, compared to vertebrate organisms, one of the significant weaknesses of cell culture toxicity assays is their inability to simulate in vivo conditions and the non-coherent interpretation of results. Therefore, animal models should be constructed to understand how these materials’ toxicity might affect living tissues.

 In the absence of in vivo studies comparing bioceramic RCSs, this study was conducted to evaluate the cytotoxicity of four bioceramic RCSs, namely GuttaFlow Bioseal, MTA Fillapex, CeraSeal Bioceramic, and iRoot SP root canal sealer using zebrafish embryos.

## Methods

###  Zebrafish husbandry and egg production

 The wild-type zebrafish of *Danio rerio* was used to produce embryos. Ten adult zebrafish were maintained in each recirculating water tank at the Central Research and Animal Facility. The zebrafish were fed flake food (TetraMin^TM^ flakes; Tetra, Melle, Germany) twice daily. The water was maintained at 27 ± 0.2 °C, the room at 24 ± 0.2 °C, and the fish was kept in a 14:10 h dark-to-light cycle.

 Zebrafish aged 6‒24 months were chosen for egg production. Adult females and males, at a ratio of 2:1, were kept in three acrylic tanks for breeding with a continuous recirculation system, each under a 12:12 hours dark-to-light cycle to ensure enough eggs were available the following morning. Each tank contained two females and one male fish. Transparent eggs appeared at the bottom of the breeding tank upon spawning. Eggs were collected and placed in the E3 solution. Unfertilized eggs were identified using an inverted microscope at × 10 magnification and discarded.

###  Preparation of E3 stock solutions for zebrafish embryos

 The one-liter 50X dilution of E3 stock solution consisted of the following chemicals in distilled water: 5.0 mM (14.6 g) sodium chloride (NaCl), 0.17 mM (0.65 g) potassium chloride (KCI), 0.33 mM (2.20 g) calcium chloride (CaCl_2_), and 0.33 mM (4.05 g) magnesium sulfate (MgSO_4_). Hydrochloric acid (HCl, 1.0 N) and sodium hydroxide (NaOH, 1.0 N) were used to adjust the pH of the stock solution to 7.2. The 1X dilution of the E3 solution was diluted with methylene blue to protect the embryos from fungal infection. All media were autoclaved.

###  Preparation of bioceramic RCSs

 Each RCS was mixed according to the manufacturer’s guidelines and delivered into a sterilized cylindrical silicone mold with a diameter of 5 mm and a thickness of 3 mm. These RCSs were incubated at 37 °C for 24 hours in a humidified incubator with 5% CO_2_ to allow a complete setting. Each RCS was placed in a 1.5-mL Eppendorf tube containing 1500 μL of 1X dilution of the E3 solution and incubated for 24 hours to produce the extract solution. The pH value of each extract solution was determined using a pH meter.

###  Fourier transform infrared (FTIR) spectroscopy analysis

 The functional groups of bioceramic RCSs were characterized using FTIR spectroscopy via a spectrometer (Spectrum Two, Perkin Elmer, the USA). The analyses encompassed four categories of samples: RCS samples untainted with the E3 solution (before immersing in E3), RCS samples immersed with E3, RCS samples suspended in E3, and the standard E3. Altogether, 13 samples were analyzed. Each sample was placed on the diamond crystal with a clean spatula and rubber dropper. The surface was carefully wiped with pure cotton wool dipped in acetone to remove foreign substances on the diamond crystal. Each sample was rapidly scanned over 30 seconds at a wavelength range of 600‒4000 cm^-1^ with a resolution of 2 cm^-1^.

###  Cytotoxicity Evaluation of Bioceramic Root Canal Sealers on Zebrafish Embryos 

 Zebrafish embryo toxicity (ZET) was analyzed based on the methods of The Organisation for Economic Cooperation and Development OECD (2013) and Makkar et al^23^ with some modifications, i.e., 96-well plates were used for one embryo instead of 24-well plates for two embryos. Fertilized eggs were examined under an inverted microscope at × 10 magnification, and the live embryos were visible 3 hours post fertilization (HPF). The embryos were randomly moved into the 96-well plates and divided into six groups as follows:

- Positive control group: embryos grown in 3,4-dichloroaniline (n = 24)
- Negative control group: embryos grown in the E3 solution (n = 24)
- Test group 1: embryos grown in GuttaFlow Bioseal extract solution (n = 16)
- Test group 2: embryos grown in MTA Fillapex extract solution (n = 16)
- Test group 3: embryos grown in CeraSeal bioceramic extract solution (n = 16)
- Test group 4: embryos grown in iRoot SP extract solution (n = 16)

All the plates were incubated at 28 ± 1 °C with a 14:10 hours light-to-dark cycle. Embryos were examined at 24, 48, 72, and 96 HPF, representing a change from one developmental phase to another. Embryonic images were captured at each HPF using a digital camera linked to the inverted microscope. Embryonic abnormalities of each test group were compared against the negative control. The same procedure was repeated three times. Each treatment used 112 zebrafish embryos; the total sample size was 336. Embryos were evaluated for their mortality, survival, and hatching rates using the following formulae:


$$\begin{array}{l} Survival\;rate\left(\%\right)\text{=}\frac{Number\;of\;alive\;embryos\;after\;96\;hpf}{Total\;number\;of\;embryos}\times \text{100}\\ Mortalilty\;rate\left(\%\right)=\frac{Number\;of\;dead\;embryos\;after\;96\;hpf}{Total\;number\;of\;embryos}\times \text{100}\\ Hatching\;rate\left(\%\right)=\frac{Number\;of\;hatched\;embryos}{Total\;number\;of\;embryos}\times \text{100} \end{array}$$

###  Statistical analysis

 Data were analyzed using SPSS 25.0. The Kruskal-Wallis and pairwise comparisons were performed to test if mortality, survival, and hatching were significantly different at a significance level of 0.05.

## Results

###  FTIR spectroscopy of bioceramic RCS

**Figure 1:**
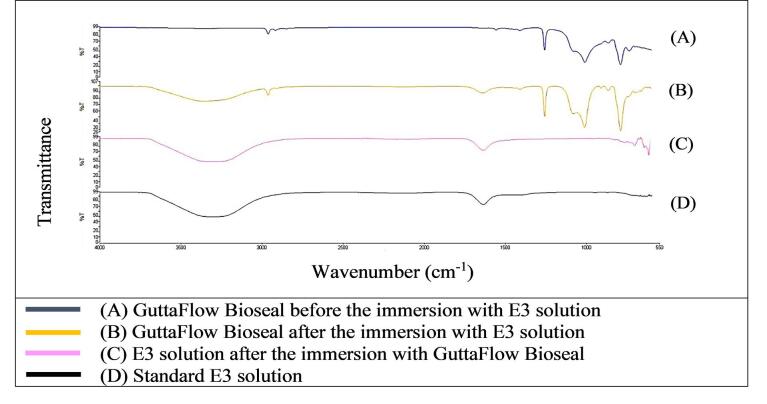
FTIR spectra of GuttaFlow Bioseal from 550 to 4000 cm^-1^

**Figure 2:**
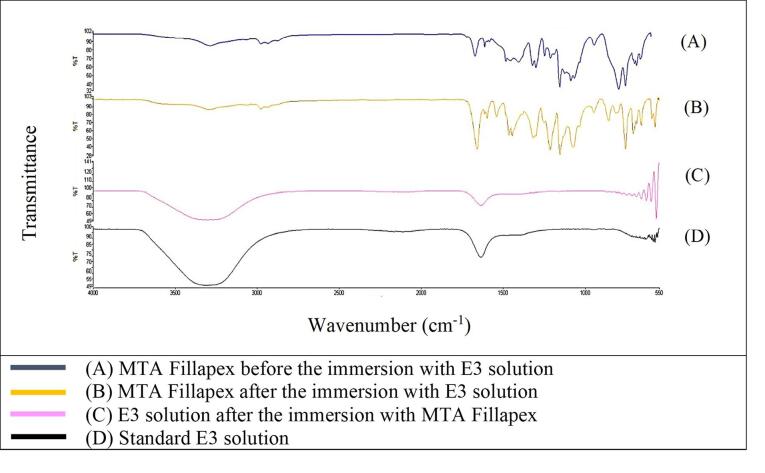
FTIR spectra of MTA Fillapex from 550 to 4000 cm^-1^

**Figure 3:**
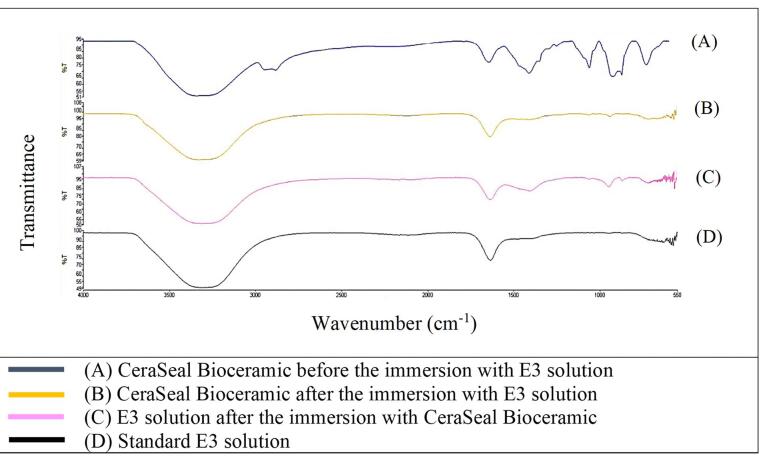
FTIR spectra of CeraSeal Bioceramic from 550 to 4000 cm^-1^

**Figure 4:**
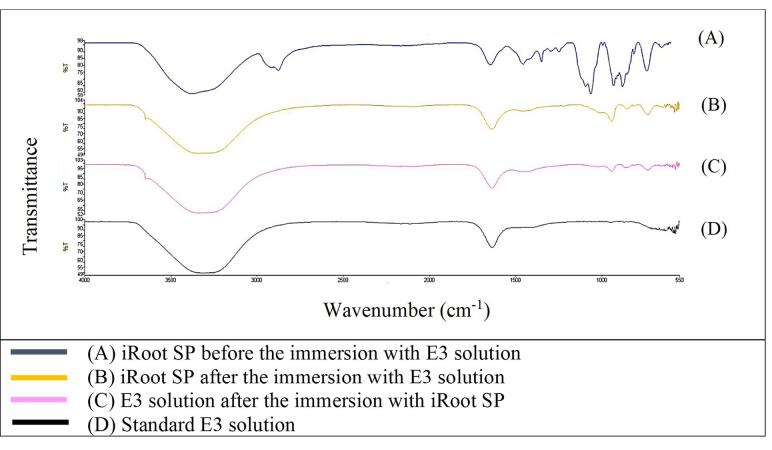
FTIR spectra of iRoot SP from 550 to 4000 cm^-1^

Figure 1 shows that the FTIR spectra of GuttaFlow Bioseal. Broad adsorption peaks occurred from 3000 to 3500 cm^-1^ in the GuttaFlow Bioseal sample immersed in E3 (B), the RCS sample immersed in the E3 solution (C), and the standard E3 (D). These broad peaks were attributable to O-H bonds. Figures 2, 3, and 4 show FITR spectra for MTA Fillapex, CeraSeal Bioceramic, and iRoot SP, respectively. Like GuttaFlow Bioseal, these three RCSs shared similar broad adsorption peaks from 3000 to 3500 cm^-1^. However, for the FTIR spectra of GuttaFlow Bioseal (Figure 1) and MTA Fillapex (Figure 2), O-H bonds were absent in untainted RCS samples before immersion in E3 (A) because they were dry materials. Meanwhile, water was detected in CeraSeal Bioceramic and iRoot SP because these materials were in the aqueous state even before immersion in E3. Additionally, weak adsorption peaks occurred from 2000 to 2500 cm^-1^ in (C) for all the four RCSs. These weak peaks were attributable to the silicone compound, i.e., calcium silicate. However, calcium silicate was absent in the standard E3 (D). These FTIR spectra indicated that all four bioceramic RCSs released their components into the E3 solution.

###  Effect of bioceramic RCSs on mortality rate of zebrafish embryo

**Figure 5:**
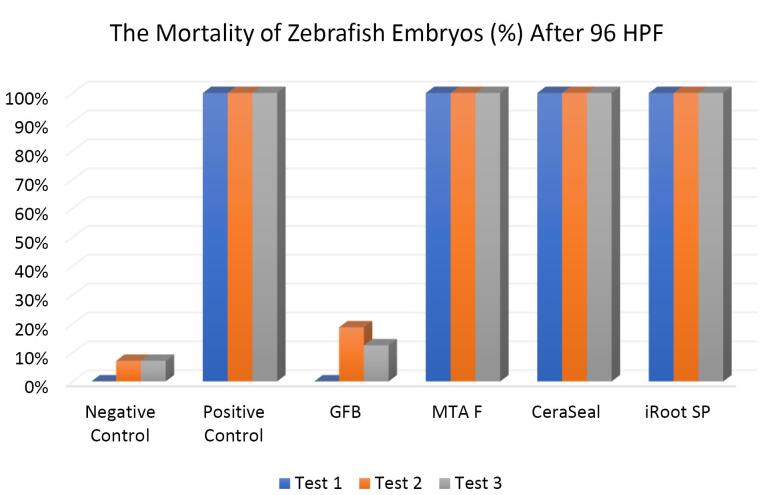
The mortality of zebrafish embryos after 96 HPF. Tests were in triplicates

Figure 5 shows the mortality rate of zebrafish embryos after 96 HPF. The negative control yielded less than 10% mortality, while the positive control had 100% mortality. GuttaFlow Bioseal showed a mortality rate relatively similar to the negative control (< 20%). Meanwhile, the positive control, MTA Fillapex, CeraSeal Bioceramic, and iRoot SP showed 100% mortality via coagulation of the embryos as early as 24 HPF. Mortality was recorded since embryos died during the development of fertilized eggs (2‒4 HPF) to 96 HPF upon exposure to bioceramic RCSs.

 The Kruskal-Wallis test showed significant differences in the mortality of zebrafish embryos between all the bioceramic RCSs and the negative control (*P* < 0.05) except for GuttaFlow Bioseal. Pairwise comparison showed that the mortality of zebrafish embryos developed in GuttaFlow Bioseal was significantly different from that of MTA Fillapex, CeraSeal Bioceramic, and iRoot SP (*P* < 0.05).

###  Effect of bioceramic RCSs on survival of zebrafish embryo

**Figure 6:**
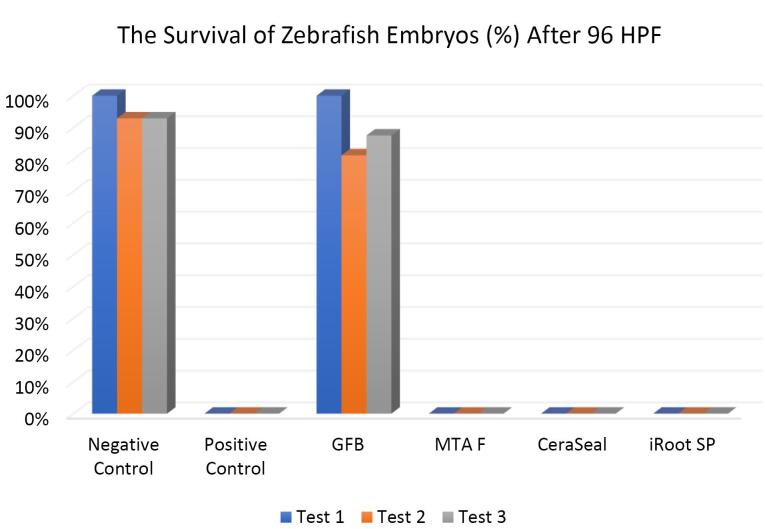
The survival of zebrafish embryos after 96 HPF. Tests were in triplicates

Figure 6 shows that the survival of zebrafish embryos at 96 HPF in GuttaFlow Bioseal was more than 80%, closely similar to the negative control. In contrast, all zebrafish embryos in MTA Fillapex, CeraSeal Bioceramic, and iRoot SP died after 96 HPF.

 Except for GuttaFlow Bioseal, the Kruskal-Wallis test showed significant differences in the survival of zebrafish embryos between the remaining three bioceramic RCSs and the negative control (*P* < 0.05). Pairwise comparisons showed that the survival of zebrafish embryos developed in GuttaFlow Bioseal was significantly different from that of MTA Fillapex, CeraSeal Bioceramic, and iRoot SP (*P* < 0.05).

###  Effect of bioceramic RCSs on the hatching of zebrafish embryos 

**Figure 7:**
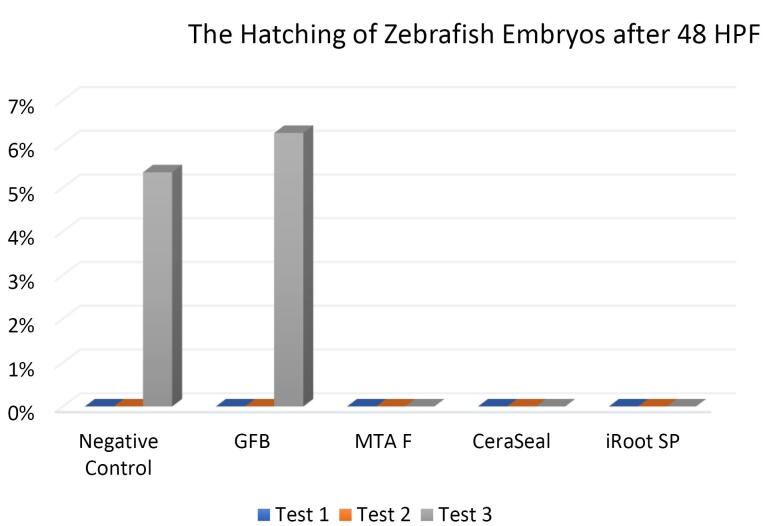
The hatching of zebrafish embryos after 48 HPF. Tests were in triplicates

**Figure 8:**
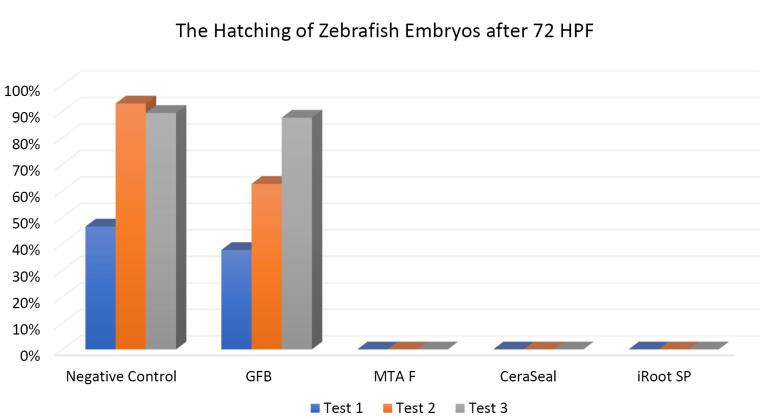
The hatching of zebrafish embryos at 72 HPF. Tests were in triplicates

**Figure 9:**
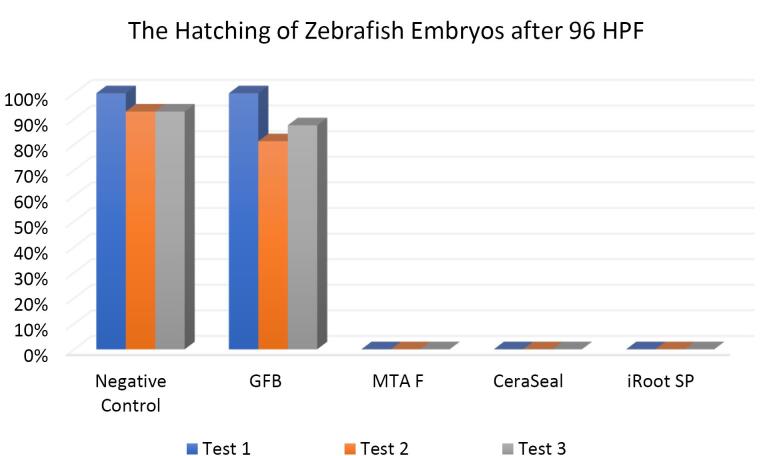
The hatching of zebrafish embryos at 96HPF. Tests were in triplicates

**Figure 10:**
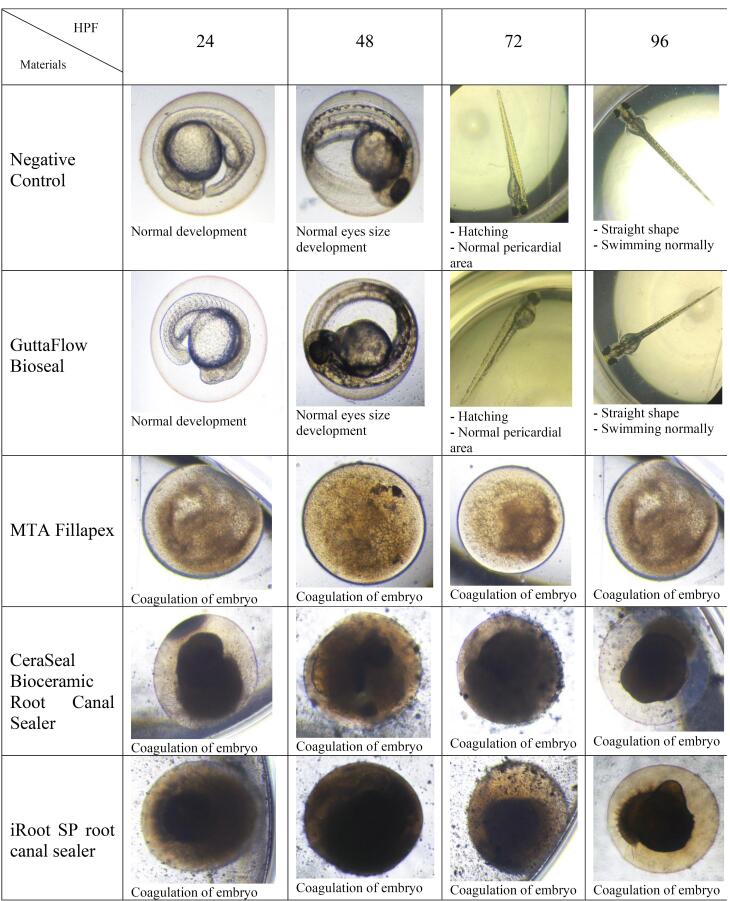
Morphological differences of zebrafish embryos after 24, 48, 72, and 96 HPF upon exposure to various bioceramic RCSs

Figures 7, 8, and 9 show the hatching of zebrafish embryos exposed to different bioceramic RCSs at 72, 48, and 96 HPFs, respectively. In the GuttaFlow Bioseal test group, the zebrafish embryos attained an average hatching of 6.25% at 48, with 62.5% at 72 HPF and 89.6% at 96 HPF. The other three RCSs showed no hatching at all the three HPFs. General morphology of zebrafish embryos upon exposure to bioceramic RCS are presented in Figure 10.

 The Kruskal-Wallis test revealed significant differences in the hatching of zebrafish embryos between the remaining three RCSs and the negative control (*P* < 0.05). Pairwise comparisons showed no significant differences in the hatching rate of zebrafish embryos between GuttaFlow Bioseal and the negative control (*P* > 0.05). Meanwhile, the hatching of zebrafish embryos developed in GuttaFlow Bioseal differed significantly from MTA Fillapex, CeraSeal Bioceramic, and iRoot SP (*P* < 0.05).

## Discussion

 Dental materials intended for clinical use must undergo a planned analysis in successive steps, including in vitro cell line cultures, in vivo studies, and preclinical studies, to determine their biocompatibility to protect patients from potential risks.^24^ The effects of these bioceramic RCSs on in vivo cytotoxicity may not be thoroughly investigated and scarcely compared between products of RCSs. In vivo cytotoxicity studies have some advantages, such as the ability to evaluate specific interactions in organisms, and are more accurate and trustworthy.^25^ However, in vivocytotoxicity of bioceramic-based root filling materials using zebrafish embryos has never been conducted before; therefore, a comparison with the present study was impossible.

 In vivo evaluation of bioceramic RCSs using animal models remains scarce. In vitro studies cannot reproduce in vivo conditions, thus yielding incoherent extrapolation of findings.^26^ Animal testing becomes the primary choice for in vivo evaluation, especially under controlled laboratory circumstances. Researchers have used many in vivo models, including rats,^27^ mice,^28^ dogs,^29^ monkeys,^30^ and ferrets.^31^ Although in vivo evaluation using animal models has a long history in toxicology and genetics, there are concerns about the cost of their maintenance and life cycle.^26^ In this respect, zebrafish (*Danio rerio*) is an effective model for toxicity screening.^32^ Zebrafish are small tropical fish that produce numerous non-adherent embryos. A single female fish can lay 200 eggs daily, making this in vivo model an economical approach for evaluating cytotoxicity.^32^ They mature rapidly and possess DNA closely similar to humans, with over 80% similarity to the human genome.^33^ Zebrafish can be kept in high numbers in compact containers.^34^ The embryos are translucent^35^ and can serve as appropriate models to investigate the morphological changes when exposed to RCSs. Nevertheless, zebrafish and mammals differ in terms of body and organ sizes.^36^ Zebrafish also have a large number of gene duplications in their genome, and their body temperatures are lower than those of mammals.^37^ Zebrafish are frequently used for toxicity analysis since they are less costly than mammalian models, such as rats, mice, and guinea pigs.^26^ Hence, researchers who use zebrafish or zebrafish embryo as model organisms should consider these key distinctions between zebrafish and humans and be aware when evaluating the results.

 The acute toxicity of bioceramic RCSs on zebrafish embryos was evaluated by the mortality, survival, and hatching at 24, 48, 72, and 96 HPF. In addition, based on the Organisation for Economic Cooperation and Development OECD, the accumulation of blood in zebrafish embryos, cardiovascular defects, coagulation of fertilized eggs, edema, lack of separation of the tailbud from the yolk sac, absence of heartbeat, failure of somite formation, and head malformations were crucial parameters for toxicity assessment.

 In this study, zebrafish embryos exposed to MTA Fillapex, CeraSeal Bioceramic, and iRoot SP root canal showed a mortality of 100%. Conversely, GuttaFlow Bioseal yielded a mortality of less than 20%. Mortality in zebrafish embryos primarily stemmed from embryonic coagulation. The differences in mortality could be attributed to the different compositions of the extract from each bioceramic RCSs and pH values. MTA Fillapex showed high mortality due to its salicylate resin matrix and high alkalinity. Meanwhile, the high mortality in CeraSeal Bioceramic and iRoot SP could be due to the zirconium oxide as a radiopacifier. In comparison, low mortality for GuttaFlow Bioseal might be attributed to its bioactive composition, which is considered to be calcium silicate particles combined with polydimethylsiloxane and gutta-percha powder. Moreover, each bioceramic RCSs have a pH value as follows: GuttaFlow Bioseal (pH = 8), MTA Fillapex (pH = 10), CeraSeal Bioceramic (pH = 13), and iRoot SP (pH = 13). Furthermore, metabolic activity in zebrafish embryonic cells appeared to be highly sensitive to pH changes, and higher pH values in MTA Fillapex, CeraSeal Bioceramic, and iRoot SP might result in oxidative stress, cytotoxicity, and hence, mortality.^38^

 The differences in the survival rates observed between MTA Fillapex, CeraSeal Bioceramic, and iRoot SP compared to GuttaFlow Bioseal might be attributed to the materials diffused through the surface of the chorion zebrafish embryos, followed by their contact with metabolic proteins.^26^ In this respect, GuttaFlow Bioseal showed less cytotoxicity than MTA Fillapex, CeraSeal Bioceramic, and iRoot SP, with zebrafish embryo survival nearly equal to the negative control, possibly due to differences in the degree of uptake of different material constituents into the embryos.^26^

 The hatching of zebrafish embryos in the GuttaFlow Bioseal group increased by 5% between 48 and 96 HPF, almost similar to the negative control. In contrast, MTA Fillapex, CeraSeal Bioceramic, and iRoot SP groups showed no hatching because the embryonic coagulation happened as early as 24 HPF. The average hatching in GuttaFlow Bioseal at 48 HPF agreed with the usual zebrafish model hatching reported in another study, i.e., between 48 and 72 HPF.^26^ Hatching rates indicate effective development of the embryo into larvae, which takes place between 48 and 72 HPF. Hatching is a crucial part of the zebrafish life cycle linked to a cascade of biochemical and physical systems. Typically, the chorion is digested during hatching by the proteolytic hatching enzyme, and the viable embryo ruptures the chorion with mechanical force to release itself.^39^ Failure to hatch in zebrafish embryos developed in the MTA Fillapex, CeraSeal Bioceramic, and iRoot SP groups might be attributable to the inability of chorion rupture following the developmental defects.^40^ Meanwhile, delayed hatching at 96 HPF might be due to the morphological defects identified in the embryos.

 However, cytotoxicity studies on these bioceramic RCSs remain scarce due to the high volume of RCSs introduced into the market, the time required to evaluate each material, and the cost of experimenting. Nevertheless, cytotoxicity evaluation informs clinicians about the safety of RCSs in endodontics.

 The limitations of this study included: (1) Comparison was performed on zebrafish embryos instead of different animal models in the same study. (2) Bioceramic RCSs were evaluated after being set rather than investigating freshly mixed state. Future studies should compare the in vivo cytotoxicity of bioceramic RCSs using various animal models.

## Conclusion

 GuttaFlow was less cytotoxic than other bioceramic RCSs; MTA Fillapex, CeraSeal Bioceramic root canal sealer, and iRoot SP root canal sealer showed comparable cytotoxicity.

## Acknowledgments

 We express our deepest gratitude to the Science Officer and Veterinary Assistant of CREAM Puteri Amirah Adib Binti Kamaruzzaman and Mohd Saiful Bin Othman for laboratory assistance. In addition, sincere thanks go to Asst. Prof. Dr. Mohamad Shafiq Bin Mohd Ibrahim for the statistical analysis

## Competing Interests

 The authors declare no competing interests.

## Ethical Approval

 Using zebrafish embryos before 6 days post fertilization (dpf) does not necessitate animal ethics committee approval. Only working with live zebrafish six days after fertilization and above requires approval from the Institutional Animal Care and Use Committee (IACUC).

## Funding

 We would like to acknowledge the Fundamental Research Grant Scheme from the Ministry of Higher Education Malaysia (FRGS/1/2019/STG07/UIAM/03/03) for the financial support of this research.
